# Establishment of an analysis model based on measurement of hepatitis B viral infection serum markers

**DOI:** 10.1186/s12879-019-3813-x

**Published:** 2019-02-18

**Authors:** Yang Guang, Li Yuzhong, Liu Hui

**Affiliations:** College of Medical Laboratory, Dalian Medical University, Dalian, 116044 China

**Keywords:** Hepatitis B, Biomarker, Mathematical model

## Abstract

**Background:**

Using serum markers of hepatitis B virus (HBV) infection, we aimed to develop a quantitative model that explains the complicated immune response to this infection.

**Methods:**

Serum samples from HBV-infected patients were randomly selected and divided into groups based on HBV-DNA positivity or negativity. Quantitative markers of HBV were measured. Formulae for Antibody index (IAb) [(anti-HBs * 1/anti-HBe + anti-HBs * 1/anti-HBc + 1/anti-HBc * 1/anti-HBe)^0.5^] and Antigen index (IAg) [(HBsAg * HBeAg)^0.5^] were introduced.

**Results:**

IAg values were statistically higher (*p* < 0.05) in the HBV-DNA-positive group than in the -negative group, but no statistically significant difference in IAb values was observed. When IAb values were > 50, IAg values were mostly < 250; when IAg values were > 250, IAb values were mostly < 50.

**Conclusion:**

IAb and IAg values can efficiently reflect the status of immune response to HBV and may be suitable for assessment of the infection process and the possible outcome of infection.

## Background

Hepatitis B virus (HBV) infection is a major public health issue worldwide [1–3]. The virus causes both chronic and acute infections. The host immune response causes both hepatocellular damage and clearance of viral antigen [4–6]. Serum markers of HBV infection might help with assessment of various issues such as prognosis [7–9].

Standard methods and reliable, commercial kits have been used to detect either HBV antigens or antibodies produced by the host. Such methods may detect hepatitis B surface antigen (HBsAg), antibody to hepatitis B surface antigen (anti-HBs), hepatitis B e antigen (HBeAg), antibody to hepatitis B e antigen (anti-HBe), or antibody to hepatitis B core antigen (anti-HBc); however, interpretation of these assays is complex [10–12]. The immune response to HBV is initiated after the virus enters the body and shows a complex relationship between the incidence and outcome of HBV, i.e. whether the patient is a disease carrier, or will develop chronic infection [7–9].

In previous assessments of anti-HBs, anti-HBe and anti-HBc responses, the data for each antibody were qualitative and the assessment for each marker was independent. Currently, quantitative serum markers of HBV infection have been used widely; however, the classical assessment rules based on qualitative test results continue to be used with quantitative results in associated analysis and studies. Therefore, we developed a new analytical model based on quantitative measurement of serum markers of HBV infection. The model explains the complicated immune response to this infection; the advantages of quantitative detection could be fully applied.

## Methods

### Data source

In total, 128 original data were collected from hospital patients with HBV infection (defined as HBsAg, HBeAg, anti-HBe or anti-HBc positive; 76 males; mean age of all patients 57.4 ± 13.6 years) at the Second Affiliated Hospital of Dalian Medical University, China. These patients were newly diagnosed by their physicians and blood samples were collected before they received antiviral treatment. There is seroconversion from an HBeAg-positive phase to an HBeAg-negative, and anti-HBe-positive phase during the natural course of infection [13]. Of 128 such patients, 23, 18 and 87 cases were respectively in HBeAg-positive, HBeAg-negative, and anti-HBe-positive phase.

### Laboratory tests

HBV markers (HBsAg, anti-HBs, HBeAg, anti-HBe, and anti-HBc) were measured using a chemiluminescent microparticle immunoassay (Cobas E601 analyzer; F. Hoffmann-La Roche Ltd., Basel, Switzerland) per the manufacturer’s protocols. Anti-HBs levels ≥10 mIU/ml were considered positive. Sample value/cut-off values (S/CO) were used as quantitative indicators for HBsAg, HBeAg, anti-HBe, and anti-HBc. S/CO ≥1.0 was defined as positive for HBsAg and HBeAg. The levels of anti-HBe and anti-HBc in the assays for these molecules are inversely proportional to S/CO; thus, S/CO ratios ≤1.0 were considered anti-HBe and anti-HBc positive.

A real-time fluorescence quantitative PCR system (Roche LightCycler 480II, Roche Ltd., Basel, Switzerland) and commercial diagnostic kits were used for the quantitation of HBV-DNA. The detection values were set at 500 IU/mL and serum samples with >500 IU/mL were considered positive for HBV-DNA.

### Establishment of quantitative model

HBsAg (a serological marker of HBV infection, both acute and chronic) and HBeAg (found in the blood when virus is present) were designated as representing the infection phase; the quantitative value for the infection phase was defined as the Antigen index (IAg). Anti-HBs, anti-HBe and anti-HBc antibodies (found after an acute infection or in chronic HBV carriers) were designated as representing the immune response phase; the quantitative value of the immune response phase was defined as the Antibody index (IAb).

**Fig. 1:**
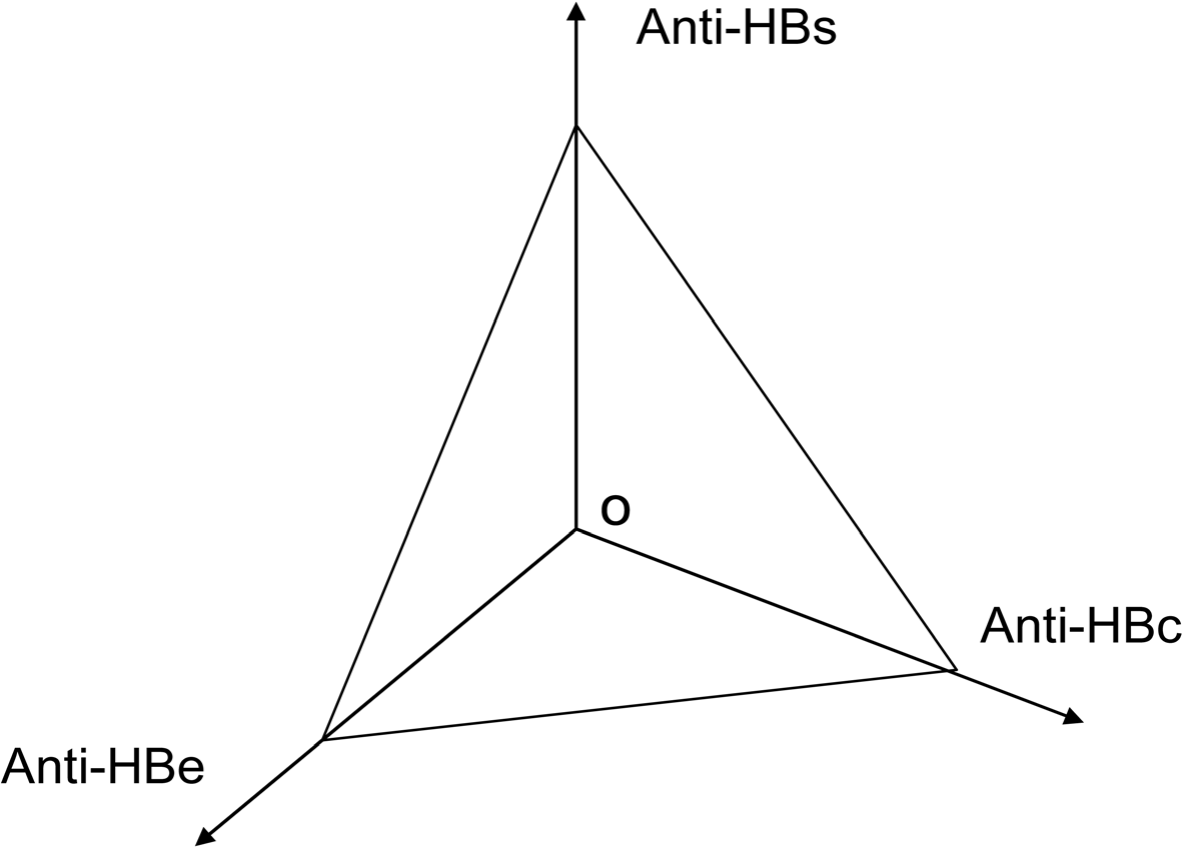
Schematic diagram of the quantitative analysis model of the immune response to hepatitis B virus (HBV)

IAb was taken as an example to explain the establishment of the model. The quantitative levels of anti-HBs, anti-HBc and anti-HBe antibodies were used to establish a three-dimensional co-ordinate system; the area of the triangle they formed was the quantitative value of infection phase (Fig. 1). The area of the triangle was calculated as:

$$ \mathrm{S}={0.5}^{\ast }\ \sin {60}^{\ast }\ \left(\mathrm{anti}\hbox{-} {\mathrm{HBs}}^{\ast }\ 1/\mathrm{anti}\hbox{-} \mathrm{HBe}+\mathrm{anti}\hbox{-} {\mathrm{HBs}}^{\ast }\ \mathrm{anti}\hbox{-} \mathrm{HBc}+\mathrm{anti}\hbox{-} {\mathrm{HBc}}^{\ast }\ 1/\mathrm{anti}\hbox{-} \mathrm{HBe}\right) $$

Note, anti-HBc and anti-HBe were determined by applying the competition method, for which (1/anti-HBe and 1/anti-HBc) should be substituted.

As 0.5 * sin60 was constant, it could be omitted in analysis.

Because the quantitative value (S) is a large number that is impractical to work with, the square root of S can be substituted as demonstrated below.

$$ IAb=\sqrt{antHBs^{\ast }1/ antiHBe+{antiHBs}^{\ast }1/ antiHBc+1/{antiHBe}^{\ast }1/ antiHBc} $$

The calculation theory for IAg was consistent with that for IAb. The formula for IAg is as follow:

$$ \mathrm{IAg}=\sqrt{{\mathrm{HBsAg}}^{\ast}\mathrm{HBeAg}} $$

### Statistical analysis

The IAg and IAb indices were not normally distributed; hence they are stated as quartile values. Difference between groups were analyzed using the Mann–Whitney *U*-test. The relationship between the IAg and IAb indices was assessed using Spearman correlation. Data were considered statistically significant when the probability of a type I error was ≤0.05. Data were analyzed using SPSS ver. 13.0 software for Windows.

## Results

Table 1 shows raw data for the HBV markers IAg and IAb in the HBV-DNA-positive and -negative groups. IAg values were higher in the HBV-DNA-positive group than in the HBV-DNA-negative group (*p* < 0.05). No significant difference in IAb level was observed between the groups.

**Table 1:** Comparison of the HBV marker between two groups of HBV-DNA

HBV marker	DNA positive (*n* = 69)			DNA negative (*n* = 59)			*p*-value
	25th	50th	75th	25th	50th	75th	
HBsAg	1593.750	5688.000	6942.750	0.452	0.520	346.500	< 0.001
Anti-HBs	1.900	1.900	9.763	1.900	16.660	127.600	0.002
HBeAg	0.099	0.128	234.200	0.085	0.091	0.104	< 0.001
Anti-HBe	0.002	0.009	1.538	0.019	0.445	1.090	0.072
Anti-HBc	0.007	0.009	0.010	0.007	0.007	0.008	< 0.001
IAg	22.695	28.413	561.247	0.194	0.218	9.151	< 0.001
IAb	21.263	156.841	233.010	46.962	106.075	220.281	0.837

Note: Total anti-HBe and anti-HBc activity level was inversely proportional to cut-off value

**Fig. 2:**
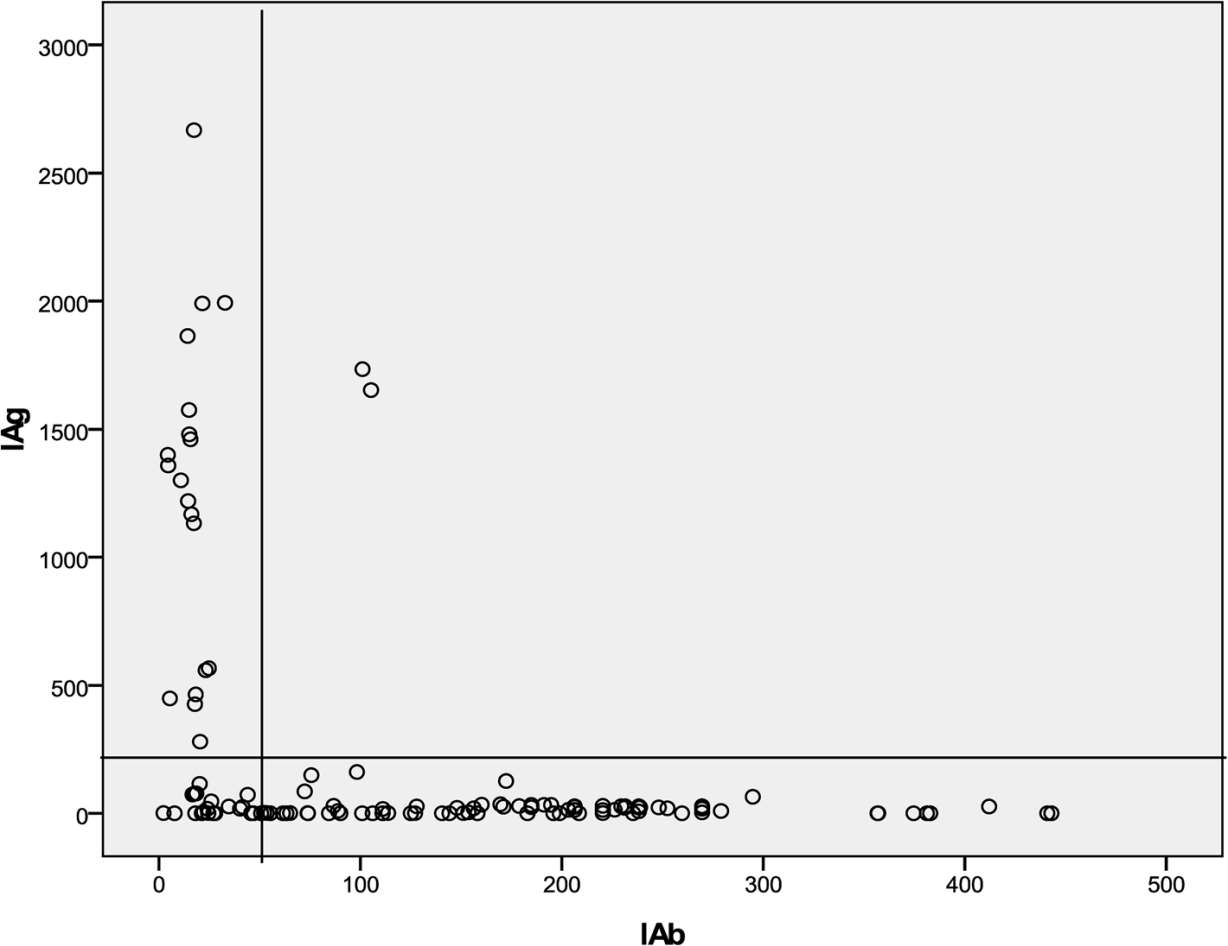
Relationship between IAg and IAb in 128 patients

Figure 2 shows the distribution of IAg and IAb in patients (x-axis, IAb values; y-axis, IAg) as a scatter plot to observe the relationship between IAg and IAb. As shown in Fig. 2, when the values of IAb were greater than 50, the values of IAg were mostly less than 250; when the values of IAg were over 250, the values of IAb were mostly less than 50. Levels of HBV-DNA in groups of scatter plot are shown in Table 2.

**Table 2:** Levels of HBV-DNA in different groups

Groups	N	HBV-DNA		
		25th	50th	75th
Positive IAb (cutoff = 50)	88	< 500	521	7207
Positive IAg (cutoff = 250)	21	538,000	3.2 × 10^7^	5.0 × 10^7^
Both IAb and IAg negative	21	< 500	< 500	3030
Both IAb and IAg positive	2	–	–	–

**Fig. 3:**
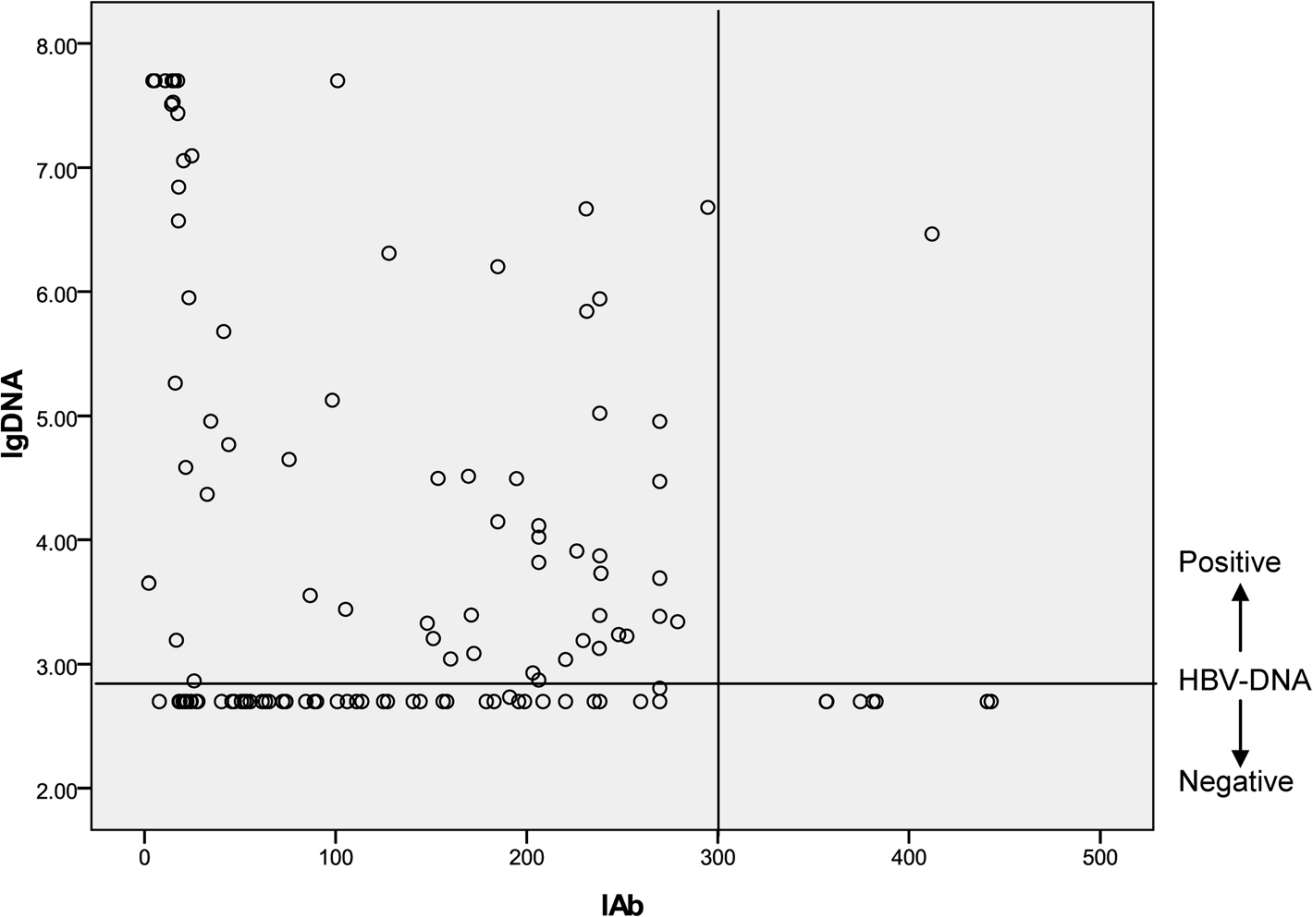
Relationship between HBV-DNA (data were logarithmically transformed) and IAb in 128 patients

Figure 3 shows relationship between IAb and HBV-DNA in 128 patients. HBV-DNA (lgDNA) was decreased with that IAb value increased. When the values of IAb were greater than 300, the values of HBV-DNA were mostly negative.

## Discussion

Tests for HBV markers (e.g. anti-HBs, anti-HBe and anti-HBc antibodies) now provide quantitative data, so a mathematical model can be established and the information used to fully evaluate the degree of immune response to HBV. Our model (IAb and IAg), established using three quantitative antibody and two antigen tests, may be a way of analyzing and studying different outcomes of HBV infection. Our results showed that when IAb values were more than 50, IAg values were mostly less than 250; when IAg values were over 250, IAb values were mostly less than 50. These results indicated that increased immune response to HBV can inhibit virus proliferation, and that the mathematical model and the cutoff values were valid in efficiently reflecting viral infection.

Although accurate quantification of HBV can be conducted in infected patients using molecular biological methods, IAg and IAb are important indexes of the extent of in vivo viral proliferation; these two tests (for HBV DNA or serum biomarkers) are related but different [14–16]. HBV infection causes immunologically-mediated damage [17–20], therefore IAg and IAb are directly related to HBV pathogenesis. No significant difference in IAb was observed between the HBV-DNA-positive and -negative groups, suggesting an important role of the immune response to HBV in pathogenicity and recovery from HBV infection.

Our results also showed that IAg were mostly negative with IAb values > 50 and the values of HBV-DNA were mostly negative when the values of IAb increase > 300, implying that risk of infectivity to other people decreased with the increase of IAb. Therefore, the new laboratory parameters IAb and IAg may be suitable tools to assess infection state during the natural course of HBV infection and further use of these data in the prediction of HBV infection outcome. We suggest that both IAb and IAg negative status (IAb < 50, IAg < 250) implies an early stage in an infection; IAg-positive (IAg > 250) implies a higher proliferation of the virus; IAb-positive (IAb > 50) implies a stage in loss of tolerance to infection and immune response to HBV; IAb > 300 implies a stage of recovery from an infection; both IAb and IAg positive are rare.

One limitation of this study is that more sensitive test was not used to better categorize HBV-DNA negative/positive patients. Howerver, the main purpose of this study was to establish a new index for assessment of the HBV infection process; our HBV-DNA result from a real-time fluorescence quantitative PCR system could be accepted for this purpose. In the future, relationship of IAb and IAg should be observed with more parameters, such as the elevation of liver enzymes and HBV-DNA with using higher sensitive system.

## Conclusion

IAb and IAg values can reflect efficiently immune response status to HBV and may be suitable for assessment of the infection process and the possible outcome of the HBV infection. The detailed clinical significance of IAb and IAg should now be determined by performing studies on various relevant areas of interest related to this disease.

## Abbreviations

anti-HBc: Antibody to hepatitis B core antigen
anti-HBe: Antibody to hepatitis B e antigen
anti-HBs: Antibody to hepatitis B surface antigen
HBeAg: Hepatitis B e antigen
HBsAg: Hepatitis B surface antigen
HBV: Hepatitis B virus
IAb: Antibody index
IAg: Antigen index
